# Empagliflozin ameliorates liver fibrosis in NASH rat model via targeting hepatic NF-κB/SOX9/OPN signaling and osteocalcin level

**DOI:** 10.1007/s00210-023-02826-6

**Published:** 2023-11-14

**Authors:** Mohamed M. Elseweidy, Abd El-Monem Ali, Sara M. Hassanin, Yasmin K. Mahmoud

**Affiliations:** 1 Biochemistry Department, Faculty of Pharmacy, Zagazig University, Zagazig, 44519 Egypt; 2 Pathology Department, Faculty of Veterinary Medicine, Zagazig University, Zagazig, 44519 Egypt; 3 Zagazig University Hospitals, Zagazig University, Zagazig, Egypt

**Keywords:** Empagliflozin, Non-alcoholic steatohepatitis, Liver fibrosis, SOX9, OPN, Osteocalcin

## Abstract

Non-alcoholic steatohepatitis (NASH) may be associated with tissue fibrotic changes and can be treated via different therapeutic tools which may however either initiate weak or long-term side effects that minimize its use. Empagliflozin (EMPA) is an oral anti-diabetic drug which has characteristic effects during hepatic steatosis regarding lipid accumulation and insulin resistance. In this study, we aimed to investigate an additional mechanism through which EMPA can exert and potentiate its anti-inflammatory and anti-fibrotic effects in NASH rat model. Male Wistar albino rats fed on high fat diet (HFD) and 20% fructose in drinking water for 18 weeks and received EMPA (30 mg/kg/day, orally) starting from week 11. Body and liver weights, homeostatic model assessment of insulin resistance (HOMA-IR), lipid profile, liver function tests, other biochemical and histological parameters were determined. HFD joined with fructose intake significantly increased body and liver weights, HOMA-IR value, hepatic inflammatory and fibrotic markers, liver transaminases, hepatic expression of nuclear factor-kappa B (NF-κB), sex determining region Y box 9 (SOX 9), and osteopontin (OPN) with significant decrease in hepatic osteocalcin (OCN). Intense hepatic lesions with severe microsteatosis and deposition of collagen fibers were clearly observed. Effectively, EMPA restored the normal liver functions, downregulated hepatic inflammatory cytokines, NF-κB, SOX 9, OPN, and increased OCN level. These results highlight another pathway illustrated the anti-fibrotic effects of EMPA against liver fibrosis probably through downregulation of NF-κB/SOX 9/OPN signaling along with upregulation of hepatic OCN which may potentiate the valuable anti-inflammatory and anti-fibrotic effects of EMPA.

**Graphical abstract:**

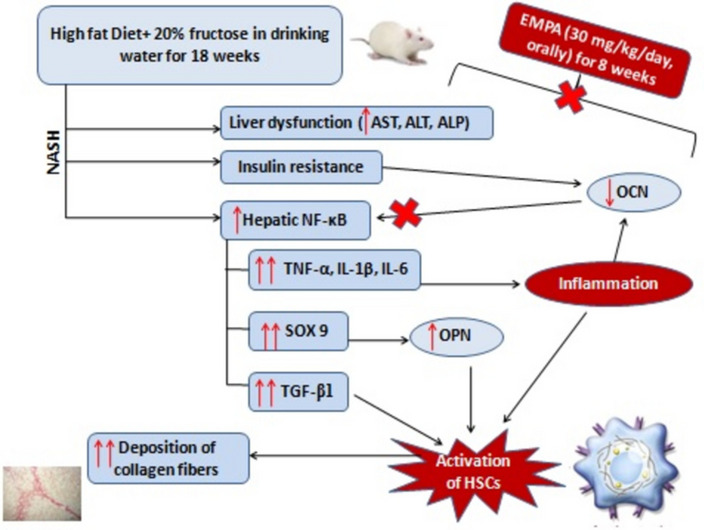

## Introduction

Non-alcoholic steatohepatitis (NASH) represents a progressive form of fatty liver disease (FLD) which is characteristically prevalent all over the world. It is characterized by steatosis, lobular inflammation, ballooning, hepatocyte injury, leading to rapid cirrhosis progression and hepatocellular carcinoma (Wong et al. 2018, Gariani and Jornayvaz 2021). Despite the growing public health impacts of NASH, its treatment options are limited.

Several factors are contributing in the initiation and progression of NASH such as insulin resistance (IR), dyslipidemia, inflammation, and oxidative stress (Attia et al. 2021). Many epidemiological studies indicated a strong link between intrahepatic fat accumulation, consumption of saturated fats, and simple sugar intake specifically fructose. These studies reported that high fructose intake can initiate hepatic steatosis along with intracellular phosphate depletion in turn NASH development (Lee et al. 2015, Takahashi et al. 2015).

Generally, excessive caloric intake either from high fat diets (HFDs) or sugar-sweetened beverages rich in fructose is mostly stored in adipose tissue. This leads to adipocyte hypertrophy, hyperplasia, free fatty acid (FFA) flux, and its hepatic accumulation. IR in addition activates fatty acid (FA) flow to the liver, enhanced hepatic lipogenesis, lipolysis, and inhibited FFA esterification. This may induce an imbalance state between synthesis/input against oxidation/exportation of hepatocellular fat (Fujii et al. 2020) which lead to excessive intrahepatic FA accumulation, activation of hepatic stellate cells (HSCs) and eventually hepatic steatosis development (Marra and Svegliati-Baroni 2018).

Once the HSCs are activated, it promotes liver fibrosis via stimulating certain profibrotic factors such as transforming growth factor-β1 (TGF-β1) and sex determining region Y box 9 (SOX 9) (Wobser et al. 2009). The latter is responsible for producing osteopontin (OPN), collagen I and also regulating the expression of other extracellular matrix (ECM) proteins (Fu et al. 2022).

Osteocalcin (OCN) is a well-recognized bone matrix protein, mainly originates from osteoblast and smooth muscle cells (Booth et al. 2013). The uncarboxylated OCN form (ucOCN) plays an important role in regulating energy metabolism and glucose homeostasis through improvement of insulin sensitivity and glucose uptake in skeletal muscles (Kirk et al. 2020). Several studies have documented a negative correlation between serum OCN and the liver functional enzymes as well as inflammatory markers which may render OCN a potential target for managing metabolic disorders (Yilmaz et al. 2011, Bador et al. 2016).

Empagliflozin (EMPA) is a novel sodium glucose cotransporter inhibitor, a class of glucose lowering agents that exerts pleiotropic effects on non-alcoholic fatty liver disease (NAFLD). Recently, different animal and clinical studies demonstrated that treatment with EMPA ameliorated hepatic fat accumulation, hepatic IR, and improved hepatic lipid metabolism during hepatic steatosis (Hüttl et al. 2021, Hiruma et al. 2023); however, its effect on hepatic OCN is not yet studied.

Thus, the present study aimed mainly to illustrate an additional mechanism through which EMPA could exert and potentiate its anti-inflammatory and anti-fibrotic effects using NASH rat model via targeting hepatic NF-κB/SOX 9/OPN axis and OCN. This may present a novel insight on EMPA potential against liver fibrosis during NASH state.

## Materials and methods

### Animals

Eighteen male Wistar albino rats weighing110 ± 10 g were supplied from the Experimental Animal Center of Faculty of Veterinary Medicine, Zagazig University, Zagazig, Egypt; and housed in controlled temperature (21–25 °C) and humidity (40–70%) under a 12-h light/dark cycles. Animal care and all experimental procedures were performed according to the protocols approved by the institutional committee of laboratory animal care and use of Zagazig University (Protocol # ZU-IACUC/3/F/15/2021) and in accordance with NIH guidelines for animal research.

### Drugs and chemicals

Empagliflozin (Mellitofix® 25 mg) was purchased from EVA Pharma, Egypt. Cholesterol was obtained from Alpha Chemical Company, India. Cholic acid was provided by Lobachemike, Mumbai, India. Fructose was obtained from Safety Misr, Egypt.

### Experimental design

After 1 week of adaptation, random group of rats was chosen, fed normal chow diet and tap water, and served as normal group (N, *n* = 6). Another group (12 rats) received HFD (60% fat + 1% cholesterol + 0.25% cholic acid) and 20% w/v fructose in drinking water for 10 weeks to induce NASH (Lee et al. 2015, Carreres et al. 2021). Thereafter, the NASH rats were randomly divided into two sub-groups (*n* = 6/group); one group was continued on HFD and 20% w/v fructose in drinking water for extra 8 weeks and served as NASH control group. The other group received EMPA (30 mg/kg body weight/day, orally) (Shao et al. 2019) along with HFD and fructose solution for 8 weeks.

### Body weight

Body weights were recorded before and after treatment period for all the studied groups.

### Blood sampling and tissue collection

At the end of the experimental study, rats were fasted overnight, weighed, and then killed by decapitation, blood samples were collected via retro-orbital plexus and centrifuged at 3000 × g for 15 min. Sera were collected and frozen at  − 20 °C for further biochemical assays. Intact livers were rapidly removed, rinsed with ice-cold normal saline solution, dried, weighed, and then divided into two portions. One portion was snap frozen in liquid nitrogen at  − 80 °C and then stored at  − 20 °C for subsequent analysis. The other part was fixed in 10% buffered formalin for 24 h and processed later to histological examination.

### Serum biochemical parameters

Glucose was measured freshly by quantitative enzymatic colorimetric determination using diagnostic kits provided from Spectrum kits, Germany (catalog #250002) (Mohamed et al. 2020). Insulin level was determined by solid phase enzyme-linked immunosorbent assay (ELISA) using rat Insulin Kit (catalog # ELR-Insulin, Ray Biotech, Norcross, GA). Total cholesterol (TC), triglycerides (TG) and high-density lipoprotein cholesterol (HDL-C) levels were determined colorimetrically using the commercial kits (catalog # TK41021, MX4103, and 1001095, respectively, Spinreact, Spain), we have followed exactly the manufacturer’s instructions (Mohamad et al. 2020). Friedewald equation was used to calculate the level of low density lipoprotein cholesterol (LDL-C), where LDL-C = TC − [HDL-C + TG/5] (Wilson et al. 1981). Serum aspartate aminotransferase (AST) was estimated colorimetrically (Huang et al. 2006) using diagnostic kits provided from Spectrum, Germany (catalog # 261001), while serum alanine aminotransferase (ALT) and alkaline phosphatase (ALP) were determined kinetically using kits provided from Spinreact, Spain (catalog # MD41274) and Spectrum, Germany (catalog # 217001), respectively (Abdelaziz and Ali 2014).

### HOMA-IR

Homeostatic model assessment of insulin resistance (HOMA-IR) was calculated using the formula ((fasting glucose (mmol/L) × fasting insulin (μIU/mL)))22.5] (Yin et al. 2014).

### Hepatic inflammatory cytokines, TGF-β1, and OCN contents

Liver tissue homogenate (10%) was prepared in 0.05 M phosphate buffer (pH = 7) using a polytron homogenizer at 4 °C. The homogenate was centrifuged at 10,000 × g for 20 min for removing any cell debris. The supernatant was used after that for the determination of hepatic interleukin (IL)-1β, IL-6, and TGF-β1 levels using rat ELISA Kits (catalog # SEA563Ra, SEA079Ra, and SEA124Ra, respectively, Cloud-Clone Corp, USA). Hepatic contents of OCN and tumor necrosis factor-α (TNF-α) were determined using rat ELISA kits (catalog # NBP2-68153, Novus Biologicals, and catalog # 438206, BioLegend Company, USA, respectively) following the manufacturer’s instructions (Wang et al. 2020) and (Guo et al. 2018).

### Hepatic OPN and SOX 9 gene expression

Total RNA was extracted from all enrolled samples with Direct-zol RNA Miniprep Plus (catalog # R2072, Zymo Research Corp., USA) following the manufacturer’s protocol and then quantity and quality were assessed by Beckman dual spectrophotometer (USA). cDNA was amplified by reverse transcription polymerase chain reaction (RT-PCR) by using SuperScript IV One-Step RT-PCR kit (catalog # 12594100, Thermo Fisher Scientific, Waltham, MA, USA). Then, the real time-PCR result was analyzed using an Applied Biosystem (StepOne™, Foster City, USA).The relative messenger RNA(mRNA) gene expression of hepatic OPN and SOX 9 were analyzed and normalized against the housekeeping gene β-actin using the cycle threshold (2^−∆∆ct^) method (Table 1), (Zhang et al. 2016) and (Fan et al. 2018).

**Table 1:** Primer sequence of the studied genes

Genes	Gene Bank accession #	Forward sequence	Reverse sequence
*OPN*	NM012881.2	5′-CAGTCGATGTCCCTGACGG-3′	5′-GTTGCTGTCCTGATCAGAGG-3′
*SOX 9*	NM080403.2	5′-TGGATGTCAAAGCAACAGGC-3′	5′-TGCGCTGGGTTCATGTAGGT-3′
*β-actin*	XM032887061.1	5′-ACATCCGTAAAGACCTCTATGCC-3′	5′-GTGCTAGGAGCCAGGGCAGTAA-3′

### Western blot analysis

The liver tissue was homogenized in ice-cold RIPA buffer followed by centrifugation in pre-cooled centrifuge tubes at 16,000 × g for 20 min in a 4 °C. All samples (20 μg protein) were separated on 4% sodium dodecyl sulfate–polyacrylamide gel electrophoresis and then transferred to polyvinylidene fluoride membranes. The membranes were blocked with 3% bovine serum albumin in tris-buffered saline with Tween 20 at 25 °C for 1 h. Then, the immunoblots were incubated with primary antibody solution of p65 NF-κB (sc-8008, Santa Cruz Biotechnology, CA, USA) overnight at 4 °C followed by incubation with the HRP-conjugated secondary antibody (Goat anti-rabbit IgG- HRP-1 mg Goat mab-Novus Biologicals) at 25 °C for 1 h (Gan et al. 2018). The blots of p65 NF-κB was visualized against control sample β-actin by using an enhanced chemiluminescence system (Clarity™ western ECL substrate BIO-RAD; CA, USA), and CCD camera-based imager and the analysis of image were performed by BIO-RAD chemiDoc MP imager.

### Histological examination

Liver tissues were fixed in 10% buffered formalin and then dehydrated in gradual ascending concentrations of ethanol, cleared in xylene, and embedded in paraffin wax. Five micron sections were cut using a microtome (Leica RM 2155, England, UK) and stained with hematoxylin and eosin (H&E). NAFLD activity score (NAS) system was employed for histopathological examination, and NASH can be diagnosed with NAS scoring exceeding 4 (Brunt et al. 2011). Sirius red stain was performed to detect liver fibrosis. The sections were examined using a light microscope and by an experienced morphologist, who was blinded to the different experimental groups.

### Statistics

Statistical analysis was performed using GraphPad Prism 7 (GraphPad Software, Inc., San Diego, CA, USA). One-way analysis of variance (ANOVA) followed by Tukey’s post hoc test were used to determine the level of significance. Correlations between OCN and other parameters were performed using non-parametric Pearson correlation analysis. *P* < 0.05 was considered statistically significant. All data were expressed as *mean* ± *SD*.

## Results

### Empagliflozin decreases body and liver weights and improves liver function tests

As shown in Table 2, after 18 weeks of consuming HFD and high fructose drinking water, all NASH rats displayed significant increases in both body and liver weight as compared to normal rats (*P* < 0.001). However, treatment with EMPA for 8 weeks showed remarkable decrease in body and liver weights in comparison with NASH control group (*P* < 0.001). NASH group also showed significant elevations in AST, ALT, and ALP levels as compared to normal group which markedly improved after treatment with EMPA (*P* < 0.001).

**Table 2:** Empagliflozin decreases body and liver weights and improves liver function tests

	N	NASH	NASH + EMPA
Body weight (g) before treatment	274 ± 19.01	352.2 ± 17.60*	342.3 ± 26.35*
Body weight (g) after treatment	282.8 ± 19.46	450 ± 51.69*	267.5 ± 22.98^#^
Liver weight (g)	5.91 ± 0.79	20.06 ± 0.58*	12.52 ± 1.572^#^
AST (U/L)	9.42 ± 1.56	22.43 ± 0.41*	9.67 ± 1.36^#^
ALT (U/L)	30.67 ± 4.81	113.7 ± 14.53*	35.33 ± 1.78^#^
ALP (U/L)	59.67 ± 19.69	619.8 ± 151.2*	179.7 ± 42.53^#^

The data are expressed as *mean* ± *SD*, (*n* = 6/group)

*SD* standard deviation, *n* sample size, *N* normal, *NASH* nonalcoholic steatohepatitis, *EMPA* empagliflozin (30 mg/kg body weight/day, orally for 8 weeks), *AST* aspartate aminotransferase, *ALT* alanine aminotransferase, *ALP* alkaline phosphatase

^*^ *P* < 0.001 vs. *N*, ^#^*P* < 0.001 vs. NASH

### Empagliflozin improves serum glucose and insulin levels, HOMA- IR value, and lipid profile

As illustrated in Table 3, there were considerable elevations in both serum glucose and insulin levels with a significant increase in HOMA-IR value in NASH group compared to the normal one (*P* < 0.001). Conversely, these increases were decreased significantly in EMPA-treated rats in comparison to NASH group (*P* < 0.001). Moreover, NASH rats displayed significant increases in serum levels of TG, TC, and LDL-C as compared to normal rats (*P* < 0.001) with a significant decrease in serum HDL-C (*P* < 0.01). Treatment with EMPA minimized the abovementioned parameters (*P* < 0.001) along with non-significant increase in HDL-C level compared to the NASH group.

**Table 3:** Empagliflozin improves serum glucose and insulin levels, HOMA-IR values and lipid profile

	N	NASH	NASH + EMPA
Glucose (mg/dL)	78.5 ± 10.01	130.8 ± 6.37*	79.17 ± 7.89^#^
Insulin (μIU/L)	3.07 ± 0.22	7.56 ± 0.52*	2.75 ± 0.27^#^
HOMA-IR	0.59 ± 0.09	2.44 ± 0.17*	0.53 ± 0.06^#^
TG (mg/dL)	88.98 ± 3.27	191.2 ± 11.98*	120.33 ± 10.95^#^
TC (mg/dL)	77.51 ± 4.07	115.5 ± 10.28*	95.23 ± 9.42^#^
HDL-C (mg/dL)	27.51 ± 3.38	20.33 ± 4.13^@^	23.50 ± 4.13
LDL-C (mg/dL)	32.21 ± 0.51	56.93 ± 6.31*	47.77 ± 2.83^#^

The data are expressed as *mean* ± *SD*, (*n* = 6/group)

*SD* standard deviation, *n* sample size, *N* normal, *NASH* non-alcoholic steatohepatitis, *EMPA* empagliflozin (30 mg/kg body weight/day, orally for 8 weeks), *HOMA-IR* homeostatic model of assessment of insulin resistance, *TG* triglycerides, *TC* total cholesterol, *HDL-C* high-density lipoprotein cholesterol, *LDL-C* low-density lipoprotein cholesterol

^*^P < 0.001 vs. *N*, ^@^*P* < 0.01 vs. *N*, ^#^*P* < 0.001 vs. NASH

### Empagliflozin downregulates hepatic p65 NF-κB protein expression and reduces inflammatory cytokines in hepatic tissues

As represented in Fig. 1A, hepatic tissues of NASH rats showed marked increase in p65 NF-κB expression with drastic raise in the hepatic levels of IL-1β, IL-6, and TNF-α (Fig. 1B) as compared to the normal group (*P* < 0.001). Treatment with EMPA induced significant downregulation in hepatic p65 NF-κB expression along with marked reduction in the hepatic inflammatory cytokines as compared to NASH control group (*P* < 0.001).

**Fig. 1a:**
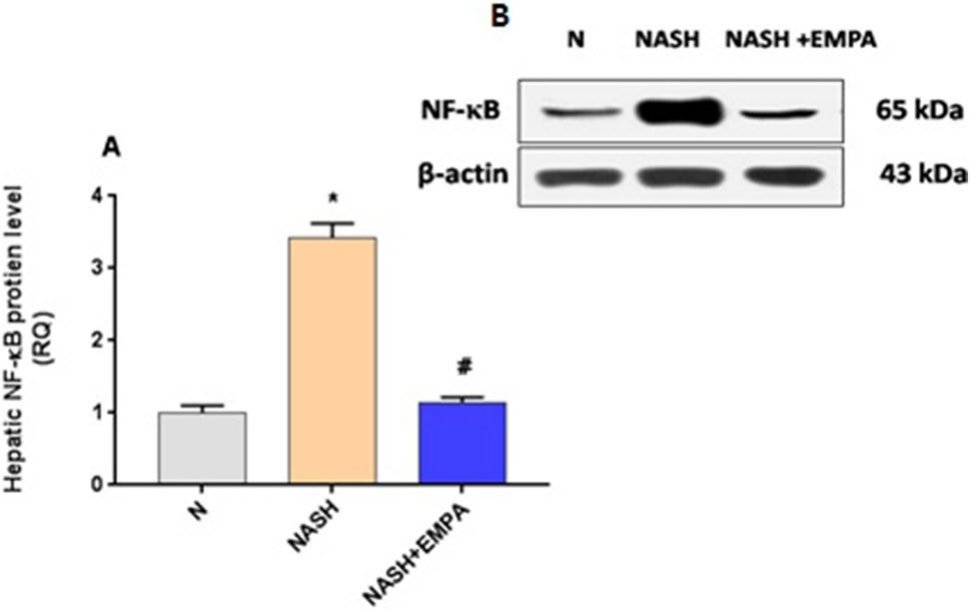
Empagliflozin downregulates hepatic p65 NF-κB protein expression. **A**) Quantitative analysis of p65 NF-κB protein expression. **B**) Representative western blots images. NF-κB: nuclear factor –kappa B, RQ: relative quantity, N: normal, NASH: nonalcoholic steatohepatitis, EMPA: empagliflozin (30 mg/kg body weight/day, orally for 8 weeks). Bars and error bars represent mean ± SD. (n=6/group). SD: standard deviation; *P< 0.001 vs. N, #P<0.001 vs. NASH

**Fig. 1b:**
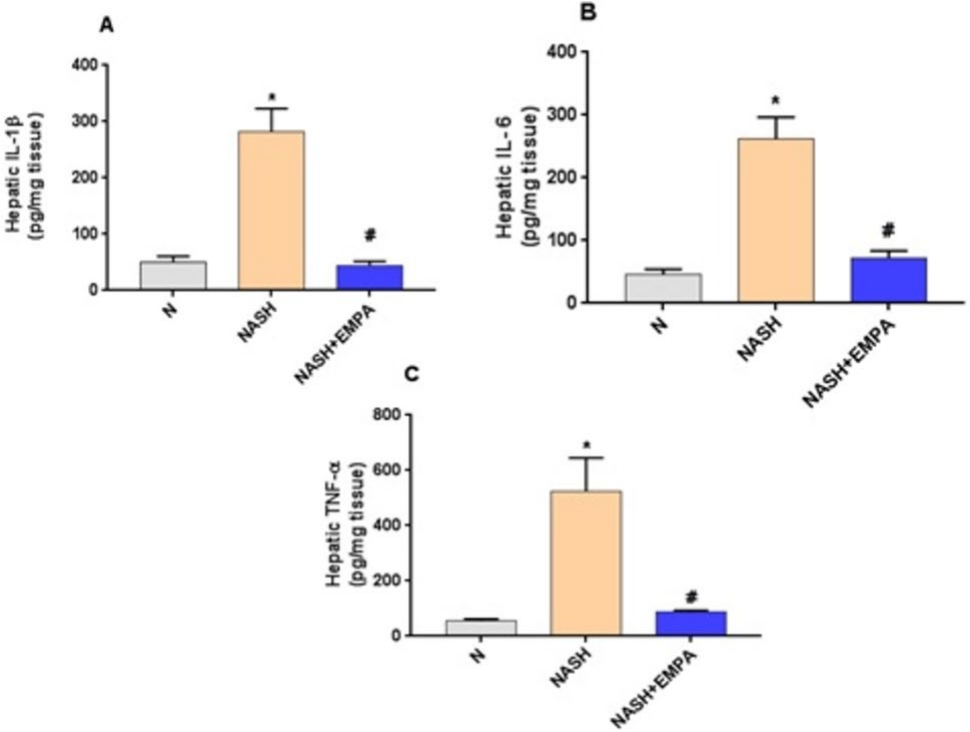
Empagliflozin reduces inflammatory cytokines in hepatic tissues. **A**) IL-1β: Interleukin-1β, **B**) IL-6, **C**) TNF-α: tumor necrosis factor-; N: normal; NASH, non-alcoholic steatohepatitis; EMPA, empagliflozin (30 mg/kg body weight/day; orally for 8 weeks). Bars and error bars represent *mean* ± *SD*. (*n* = 6/group). *SD*, standard deviation; * *P* < 0.001 vs. *N*, ^#^*P* < 0.001 vs. NASH

### Empagliflozin represses hepatic TGF-β1 and SOX 9 gene expression

**Fig. 2:**
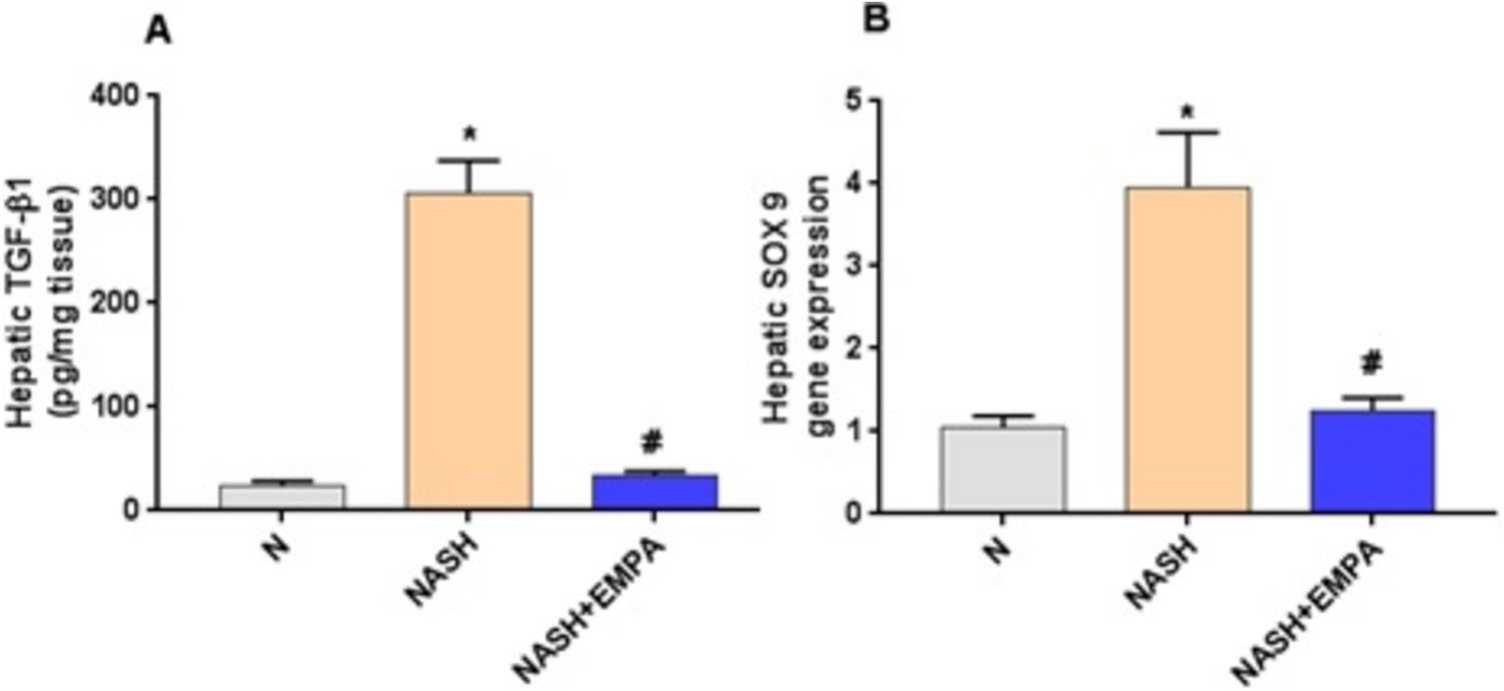
Empagliflozin represses hepatic TGF-β1 and SOX 9 gene expression. **A** TGF-β1, transforming growth factor-β1, **B** SOX 9, sex determining region Y box 9, N, normal; NASH, non-alcoholic steatohepatitis; EMPA, empagliflozin (30 mg/kg body weight/day, orally for 8 weeks). Bars and error bars represent *mean* ± *SD*. (*n* = 6/group). *SD*, standard deviation; *P < 0.001 vs. *N*, ^#^*P* < 0.001 vs. NASH

Hepatic tissues of the NASH control group demonstrated marked increase in both TGF-β1and SOX9 mRNA expression by 12.7 and 3.8 folds, respectively, compared to the normal control group (*P* < 0.001), which were repressed noticeably after treatment with EMPA (*P* < 0.001) (Fig. 2).

### Empagliflozin reduces OPN level with significant upregulation of hepatic OCN

**Fig. 3:**
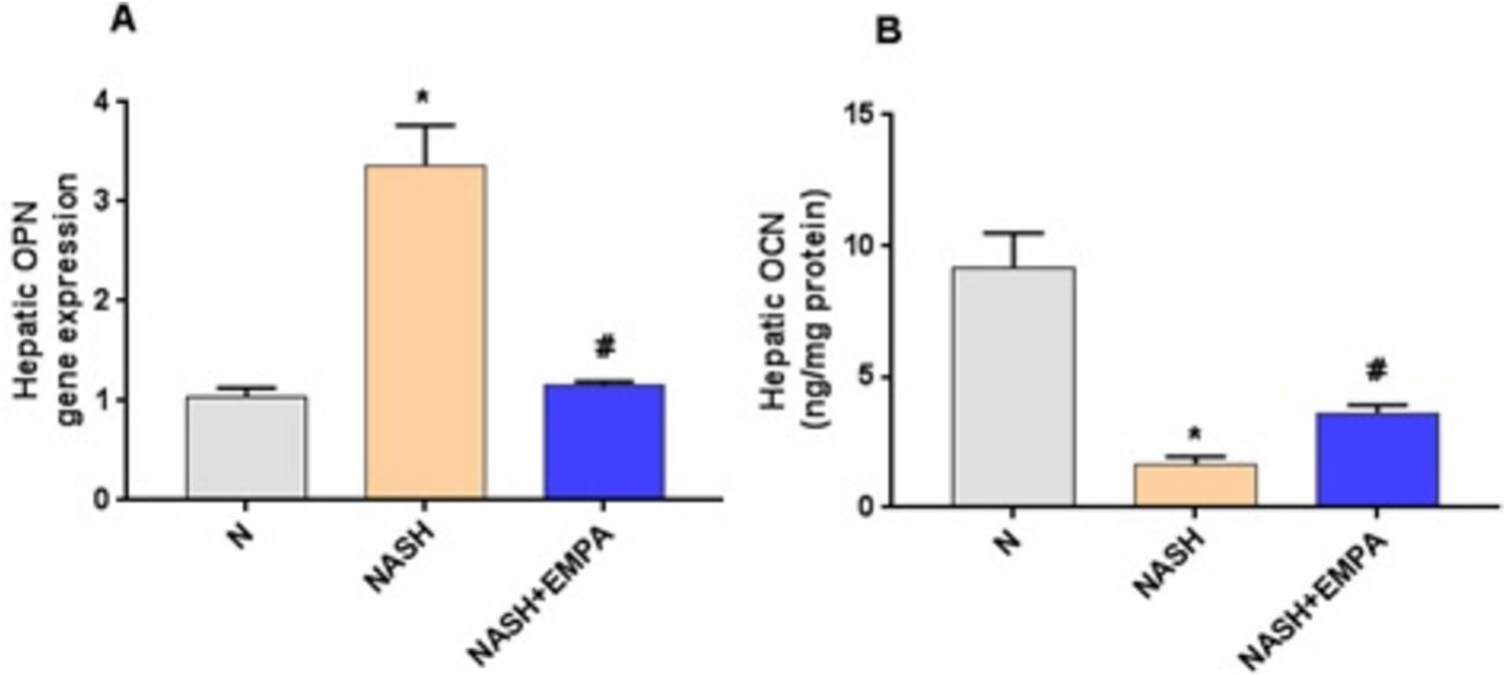
Empagliflozin reduces OPN level with significant upregulation of hepatic OCN. **A** OCN: osteocalcin, **B** OPN: osteopontin, N, normal; NASH, non-alcoholic steatohepatitis; EMPA, empagliflozin (30 mg/kg body weight/day, orally for 8 weeks). Bars and error bars represent *mean* ± *SD*. (*n* = 6/group). *SD*, standard deviation; *P < 0.001 vs. *N*, ^#^*P* < 0.001 vs. NASH

As shown in Fig. 3, NASH rats displayed a marked reduction in the hepatic content of OCN with a significant elevation in hepatic OPN level in comparison with the N group (*P* < 0.001). Interestingly, EMPA-treated group showed significant upregulation of hepatic OCN with marked decrease in hepatic OPN level as compared to NASH control group (*P* < 0.001).

### Empaglifozin ameliorates histological lesions, microsteatosis, and suppresses collagen fiber deposition

**Fig. 4:**
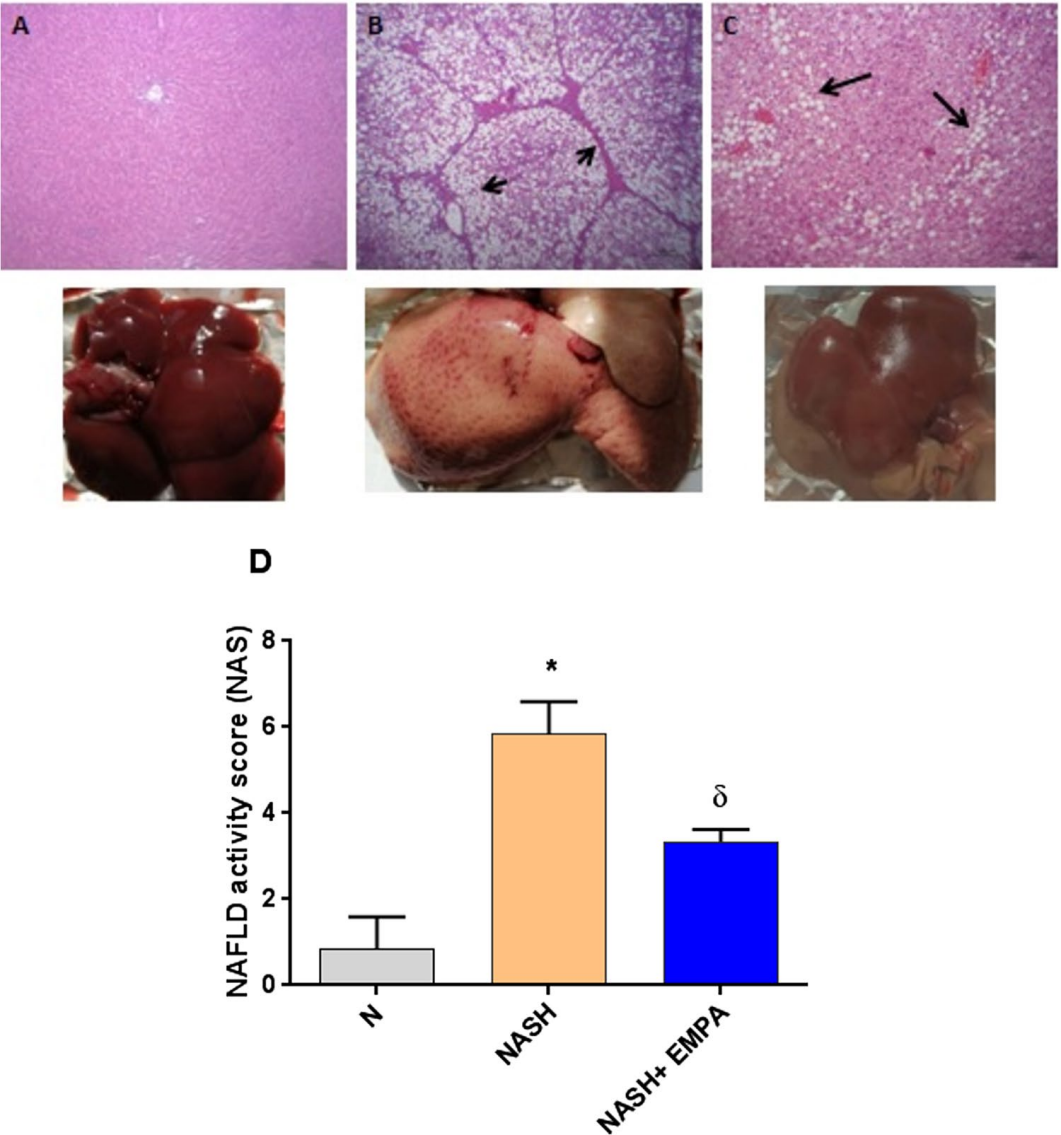
Representative photomacrographs of liver and photomicrographs of H&E stained sections of hepatic tissues from different experimental groups (H&E staining × 200, scale bar = 100 μm). **A** Normal control group showing normal hepatic parenchyma. **B** NASH control group displayed massive microsteatosis (arrow) and fibrosis (arrow head) with mild peripherilobular microsteatosis (arrow). **C** NASH + EMPA group showed moderate perilobular microsteatosis (arrow) and delicate fibrous strands (arrow head), **D** NAFLD activity score (NAS) analysis. Bars and error bars represent *mean* ± *SD*. (*n* = 3/group). *SD*, standard deviation; *P < 0.001 vs.* N*, ^δ^*P* < 0.01 vs. NASH

**Fig. 5:**
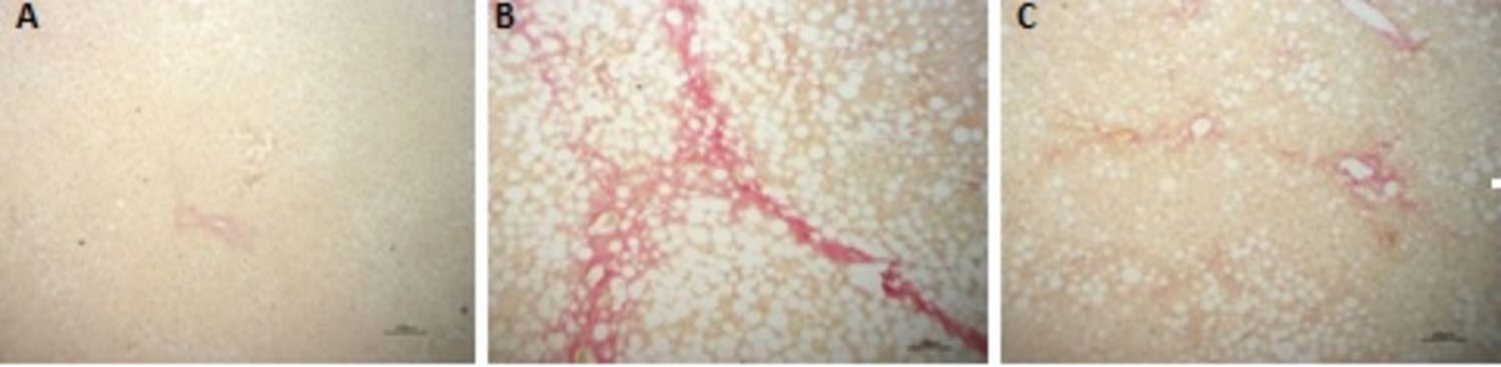
Representative photomicrographs of siruis red-stained sections of hepatic tissues from different experimental groups (Siruis red × 200, scale bar = 100 μm). **A** Normal control group showing no collagen fibers deposits. **B** NASH control group showing intense collagen fibers stained red in portal areas and interlobular tissue. **C** NASH + EMPA group showing delicate collagen deposits in portal and interlobular tissue, (*n* = 3/group)

As shown in Fig. 4; hepatic tissue of the normal group demonstrated normal histological pattern of hepatic parenchyma, hepatic cell vasculature and portal areas. NASH rats showed hepatocytes with intense lesions and severe microsteatosis varied from zonal to diffuse patterns beside portal and lobular fibrosis. Also, bile ductule proliferation was encountered. NASH group showed also a significant increase in the NAS level in comparison to the N group (*P* < 0.001). However, the EMPA-treated rats showed better improvement in lesions characterized by moderate zonal microsteatosis without delicate fibrosis, and the remaining hepatic parenchyma was apparently normal. Furthermore, the NAS level decreased significantly (*P* < 0.01) as compared with NASH control group. To evaluate the degree of fibrosis, Sirius red stain was used. As illustrated in Fig. 5, extensive collagen fiber deposition is present (stained red) and located mainly in the portal areas and interlobular tissue in the liver of NASH rats with moderate improvement in EMPA-treated rats where fine or delicate fibrous strands deposit. Type and score of lesions are summarized in Table 4.

**Table 4:** Summary of type and score of lesions

Type of lesions	N	NASH	NASH+EMPA
Microsteatosis	-	+++	+
Portal fibrosis	-	+++	++
Lobular fibrosis	-	++	-
Hyperplastic bile duct	-	++	+
Mononuclear cell infiltration	-	++	+
Acute cell swelling	-	-	-
Normal hepatic parenchyma	+++	-	++

### Biochemical correlations

**Fig. 6:**
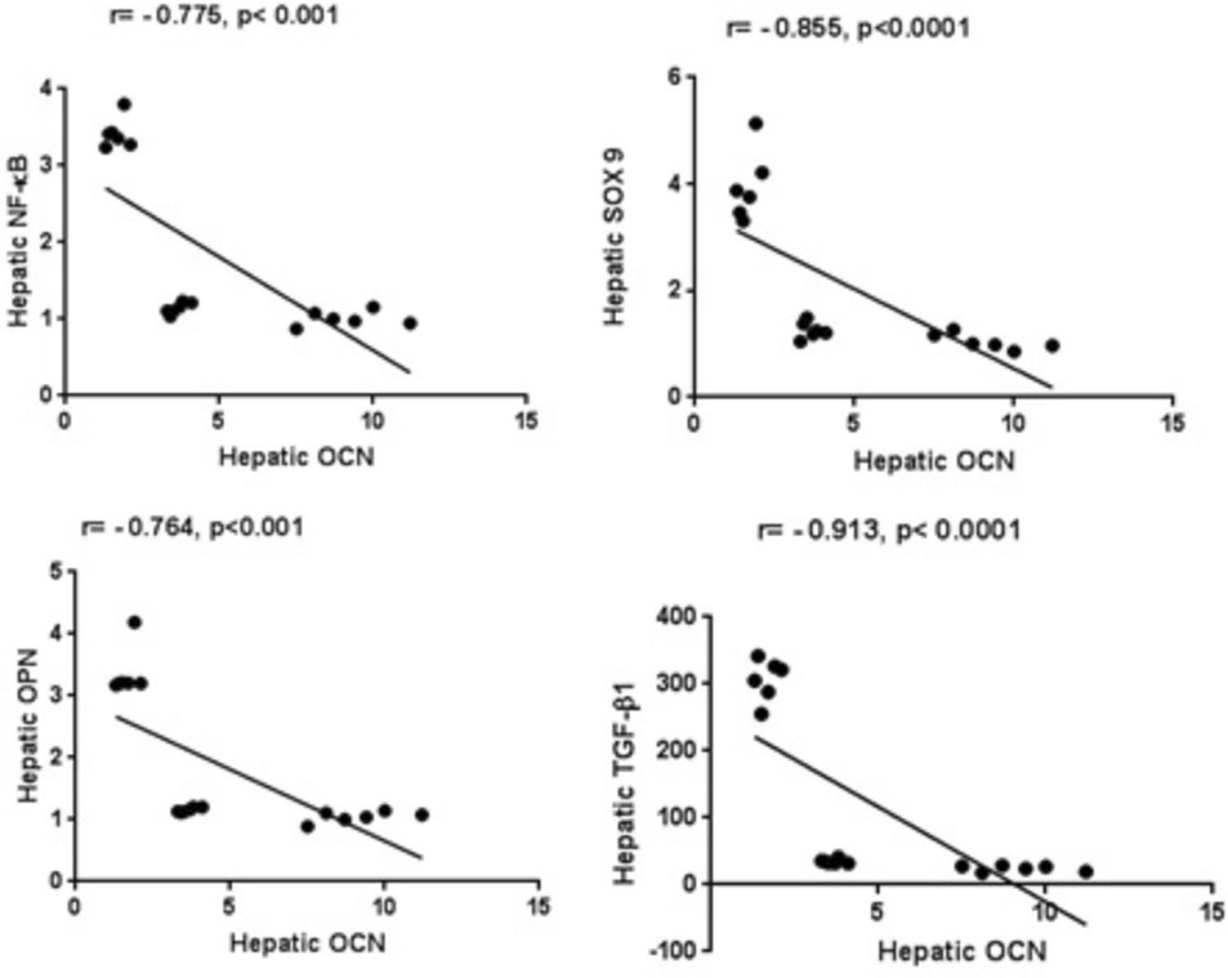
Non-parametric Pearson correlation analysis. OCN, osteocalcin; NF-κB, nuclear factor-kappa B; SOX 9, sex determining region Y box 9; OPN, osteopontin; TGF-β1, transforming growth factor- β1

Non-parametric Pearson correlation analysis was performed to explore the association between hepatic OCN and NF-κB, SOX 9, OPN, and TGF-β1 using the combined data from all the experimental groups. As demonstrated in Fig. 6, hepatic OCN is negatively correlated with NF-κB (*r* =  − 0.775, *P* < 0.001), SOX 9 (*r* =  − 0.855, *P* < 0.0001), OPN (*r* =  − 0.7647, *P* < 0.001), and TGF-β1 (*r* =  − 0.913,*P* < 0.0001).

## Discussion

The present study demonstrated that empagliflozin could probably alleviate liver fibrosis during NASH via downregulation of NF-κB/SOX 9/OPN axis and upregulation of OCN in the hepatic tissues. Empagliflozin intake daily for 8 weeks ameliorated to a certain extent liver fibrosis generally through IR improvement and decreasing hepatic inflammatory cytokines as well as TGF-β1. Importantly, empagliflozin showed a marked reduction in the hepatic NF-κB/SOX 9/OPN signaling, which led to a decrease in collagen fiber deposition along with increasing hepatic OCN content.

NASH is usually characterized by IR as a main contributor to its pathophysiology and several pharmacological options have been used for its management such as insulin sensitizers and lipid-lowering agents, and both exerted weak effects or long-term side effects that prohibited their use (Gerges et al. 2020, Godoy-Matos et al. 2020).

In the current study, rats received high fat diet and 20% fructose in drinking water for 18 weeks displayed increased body and liver weights, elevated serum glucose level, IR, disturbed lipid profile, and disturbed liver function tests. Severe microsteatosis, collagen fiber deposition and lobular fibrosis were also demonstrated and in agreement with previous reports (Lee et al. 2015, Navik et al. 2022).

Prolonged consumption of diets high in fats, cholesterol, and fructose also referred to as western diets can induce IR through an increase in de novo lipogenesis, TG levels in portal circulation and subsequently excessive hepatocyte fat accumulation. Additionally, high fat intake induces hepatic IR directly through activation of protein kinase c/Jun terminal kinase 1 which impairs the phosphorylation of both insulin receptor substrate and insulin receptor substrate-2 tyrosine (Stanhope 2016, Perla et al. 2017). Moreover, IR is joined by NF-κB activation which in turn stimulate the expression of target genes encoding inflammatory mediators, such as TNF-α, IL-6, and IL-1β (Mohamed et al. 2020). This makes NF-κB to be one of the most important regulators of liver injury and hepatic inflammation in NASH state (Incir et al. 2016, Li et al. 2018). In accordance, our data demonstrated an activated inflammatory state where elevated levels of hepatic NF-κB, IL-1β, IL-6, and TNF-α were recorded due to intake of HFD along with fructose in drinking water for 18 weeks.

The main location of NF-κB is in the cytoplasm as a complex of some inhibitory IκB proteins. Once NF-κB is stimulated, it activates IκB kinases (IKKs) that target IκBs to be degraded by the proteosome, releasing NF-κB p65 phosphorylation which allow its nuclear translocation and activating the target gene such as TNF-α, IL-1β, and IL-6(Bakovic et al. 2020).

Fibrosis represents a clear step in chronic liver diseases (CLDs), leading to severe liver dysfunction and death. Generally, it results from excessive accumulation of ECM proteins due to an imbalance between their production and degradation. During CLDs including NASH, activated HSCs represent the major ECM-producing cells (Wallace et al. 2015). Previous reports have illustrated various intracellular pathways that promote HSC activation. For instance during IR, higher insulin level may directly stimulate HSCs to proliferate and secrete type I collagen. Hepatocyte stress and death in addition can promote inflammation which activates HSCs indirectly via stimulating macrophage recruitment and secretion of pro-fibrogenic mediators such as TGF-β1 (Cai et al. 2017, Fujii et al. 2020).

Another important pro-fibrotic factor which shares progression of liver fibrosis is SOX 9. It is identified as a key transcription factor responsible for producing number of ECM proteins by the activated HSCs (Pritchett et al. 2014). Also, it is associated with the inflammatory response through regulating NF-κB pathway (Saegusa et al. 2012) additionally can activate TGF-β1 (Fan et al. 2018). Zhu et al. (2018) reported a signaling pathway involving SOX 9 that mediates fibrosis during NASH independent of hepatocellular injury. They showed that activation of hepatocyte notch by high fat, high fructose diet can induce fibrosis via increasing SOX 9-dependent OPN expression and secretion from hepatocytes. This sequentially activates the resident HSCs leading to excessive collagen deposition (Zhu et al. 2018). Our results in agreement showed a significant increase in hepatic SOX 9 and OPN gene expression along with elevated level of hepatic TGF-β1 and extensive deposition of collagen fibers in hepatic tissues in control group.

On the other hand, OCN is a non-collagenous protein, synthesized, and released mainly by osteoblasts and considered as a conventional marker of bone formation (Ducy et al. 1996). Reported studies demonstrated its ability in the uncarboxylated form to act as hormone and enhancing insulin action and secretion. This in turn can increase insulin sensitivity and improve glucose metabolism in both human and animal studies (Bulló et al. 2012, Zhou et al. 2013, Bonneau et al. 2017, Huang et al. 2017). Additionally, it promotes the expression of FA transporters and stimulates β-oxidation (Mera et al. 2016).

An association between OCN and liver diseases had been previously reported. In NAFLDs including NASH, low serum OCN level was observed in contrast to high liver enzymes, attributed to IR, and systemic chronic low-grade inflammation (Lim et al. 2021). It is well known that IR includes disorders in glucose and lipid metabolism which leads to ectopic accumulation of FFA in hepatocytes. Also, systemic low-grade chronic inflammation involves high levels of pro-inflammatory cytokines and activated immune cells that aggravates IR, leading to hepatocellular injury and fibrosis (Torre et al. 2021). Our data in agreement demonstrated a marked decrease in hepatic OCN level along with elevated liver enzymes in NASH model.

EMPA is an oral hypoglycemic drug, which represents SGLT2 inhibitor candidates in renal tissues (J Levine 2017), improves hepatic insulin sensitivity, and additionally, it decreases hepatic lipid accumulation indeed in NAFLD progression (Chehrehgosha et al. 2021, Androutsakos et al. 2022). Its beneficial effect on hepatic fibrosis may be due to its inhibition of pro-inflammatory cytokines such as IL-6 and TNF-α in the liver (Chehrehgosha et al. 2021); however, the other mechanisms involved in reducing hepatic fibrosis by EMPA remain unclear.

In this study, treating NASH rats with EMPA for 8 weeks significantly improved IR, lipid profile, and liver function tests, and downregulated hepatic inflammatory cytokines in addition to TGF-β1 via decreasing hepatic NF-κB in agreement with prior studies (Mohamed et al. 2020, Al-Wakeel et al. 2022). Interestingly, our data showed that treatment with EMPA induced significant reductions in both hepatic SOX 9 and OPN levels along with a remarkable decrease of collagen fiber deposition. It is worth to be mentioned that NF-κB shows a positive regulation on SOX9 expression by binding directly to its promoter (Sun et al. 2013) while SOX 9 stimulate OPN synthesis (Zhu et al. 2018). Therefore, the abovementioned results clarified another pathway through which EMPA can exert its anti-fibrotic effect via downregulation of NF-κB/SOX 9/OPN signaling pathway, inhibition of HSCs, and in turn the decrease in collagen fiber production.

Prior studies illustrated various protective effects of OCN against NASH which mostly are attributed to a decreased expression of pro-inflammatory genes along with activation of the antioxidant genes involved in the nuclear factor-E2-related factor-2 and NF-κB signaling pathways (Zhou et al. 2013, Du et al. 2016). OCN, in addition, can increase insulin sensitivity through adiponectin expression increase in white fat along with decreased lipid accumulation in steatotic liver (Ferron and Lacombe 2014). Zhang et al. (2020) showed that OCN may inhibit lipid synthesis, promote lipolysis in liver, and attenuate inflammatory responses in experimental mice (Zhang et al. 2020). These results render OCN to be a valuable therapeutic target, and its upregulation may be beneficial against liver fibrosis especially during NASH.

Herein, we noticed a remarkable upregulation of hepatic OCN level after treatment with EMPA which provides another valuable effect of EMPA during NASH treatment. It is important to mention that p65 NF-κB has a negative regulatory effect on OCN gene expression (Tarapore et al. 2016). Therefore, here, the downregulation of hepatic NF-κB by EMPA effectively stimulated the upregulation of OCN in hepatic tissues.

Collectively, the present study demonstrated an additional pathway regarding the anti-inflammatory and anti-fibrotic potential of EMPA in the NASH model. This was achieved through downregulation of NF-κB/SOX 9/OPN axis along with the upregulation of hepatic OCN level. Daily EMPA intake for 8 weeks efficiently ameliorated IR and improved lipid profile and liver function tests associated with decreased hepatic inflammatory cytokines and TGF-β1. This was achieved through downregulation of NF-κB/SOX 9/OPN signaling pathway and in turn inhibition of HSCs and decreased collagen fiber deposition in hepatic tissues. Important finding is the significant upregulation of hepatic OCN content due to the inhibition of NF-κB signaling which enhanced subsequently the anti-inflammatory and anti-fibrotic effects of EMPA. However, further preclinical and clinical studies are required to validate our results.

## Study limitations

One limitation of the current study is that we did not include in our experimental design a group of normal rats treated with EMPA. This group could have helped us to identify the potential benefits of using EMPA against the occurrence of steatohepatitis, in particular NASH, even in the absence of HFD or fructose in drinking water.

## Data Availability

Data are available upon request to the corresponding author.
